# Application of mPEG-CS-cRGD/*Bmi-1RNAi*-PTX nanoparticles in suppression of laryngeal cancer by targeting cancer stem cells

**DOI:** 10.1080/10717544.2023.2180112

**Published:** 2023-02-21

**Authors:** Xiaoyan Xu, Tianhao Zhou, Xudong Wei, Xuelian Jiang, Jiyan Cao

**Affiliations:** a Department of E.N.T, Gansu Provincial Hospital, Lanzhou, P.R. China; b The First School of Clinical Medicine, Gansu University of Chinese Medicine, Lanzhou, P.R. China; c The First School of Clinical Medicine, Lanzhou University, Lanzhou, P.R. China; d Department of Clinical Medicine, Ningxia Medical University, Yinchuan, P.R. China

**Keywords:** laryngeal cancer, cancer stem cells, nanoparticles, Bmi-1, cRGD peptide, paclitaxel

## Abstract

Although surgery-based comprehensive therapy is becoming the main approach to treat laryngeal cancer, recurrence, metastasis, radiotherapy resistance and chemotherapy tolerance are still the main causes of death in patients. Targeted inhibition of laryngeal cancer stem cells has been considered as the consensus to cure laryngeal cancer. Our previous study has confirmed proto-oncogene *Bmi-1* as *a* key regulator for self-renewal of laryngeal cancer stem cells. Targeted knockdown of *Bmi-1* gene effectively inhibited the self-renewal and differentiation of laryngeal cancer stem cells, leading to the promoted sensitivity to chemotherapy including paclitaxel. However, due to off-target effects and quick degradation of the naked *Bmi-1*-RNAi small RNA oligo by nuclease in body fluids, it is urgently needed to develop a tumor-targeted delivery system with a protective shell. In this study, we designed and synthesized cRGD peptide-modified chitosan-polyethylene glycol slow-release nanoparticles (mPEG-CS-cRGD/*Bmi-1RNAi*-PTX) containing *Bmi-1RNAi* siRNA oligo and paclitaxel, which showed spherical in shape, 200 nm diameter in size, low cytotoxicity, strong DNA wrapping, resistance to nuclease degradation and high transfection efficiency to cells. Functional analysis indicated significant suppression of cell proliferation and migration and induction of apoptosis by the nanocomplex in laryngeal cancer cells *in vitro*. By application to the mouse model with laryngeal cancer, the nanocomplex inhibited tumor growth significantly *in vivo*. In addition, cRGD peptide, paclitaxel and *Bmi-1* siRNA in the nanoparticles showed synergistic effects to suppress laryngeal cancer stem cells. In conclusion, this study not only developed a laryngeal tumor-targeted chemotherapeutic system, but also demonstrated a *Bmi-1* RNAi-based chemotherapeutic strategy to inhibit cancer stem cells, having strong potential to treat laryngeal cancer patients suffering therapy resistance and/or tumor recurrence.

## Introduction

Laryngeal cancer is the second most common cancer of the respiratory tract after lung cancer, and ranks second among head and neck squamous cell carcinomas (HNSCC) (Liu et al., 2018). It is the second most common cancer of the respiratory tract after lung cancer. The traditional treatment of laryngeal cancer is mainly a combination of surgical procedures, but recurrence, metastasis, resistance to radiotherapy and tolerance to chemotherapy are still the main causes of death, and there is an urgent need to develop new treatments from a new perspective. In recent years, the theory of Cancer stem cells (CSCs) suggests that there are very few CSCS with infinite proliferation and multi-differentiation potential in tumors, which are the seeds of tumors and the source of infinite proliferation, recurrence and metastasis (Ayob & Ramasamy, 2018). CSCs have been successfully isolated from a variety of head and neck tumors (Chen et al., 2020). CD133 is a 5th transmembrane glycoprotein with a molecular weight of 120 KD and is considered a marker of normal hematopoietic stem cells (Arndt et al., 2013). The study concluded that CD133^+^ cells have higher self-renewal, proliferation, colony formation and in vitro tumourigenic capacity than CD133^-^ cell population in laryngeal Hep-2 cells (Zhou et al., 2007). Some studies have reported that C-myc, Nanog homeobox (Nanog), POU class 5 homeobox 1 pseudogene 4 (oct-4), SRY-box transcription factor 2 (Sox-2) are all CSCs-related cytokines, among which the core transcriptome composed of Nanog, Oct-4 and Sox-2 is involved in the self-renewal of CSCs and is a key influencing factor in the transcription and translation of CSCs-related genes (van Schaijik et al., 2018). A similar role for C-myc, Nanog, Oct-4 and Sox-2 has been reported in HNSCC (Singh et al., 2021).

Nanoparticles (NPs) are micron-sized colloidal particles, about 1 0 ∼ 100 nm in size, which can be classified into organic nanoparticles, inorganic nanoparticles, metal nanoparticles, polymer nanoparticles, etc. according to their chemical species. Due to NPs are adsorbent and biologically active to the target tissues while avoiding phagocytosis by the endogenous phagocytic system, thus can be effective in the transit process protects the drug and can be used as a carrier for potential drugs and diagnostic agents, effectively targeting to the target cells, usually tumor cells, for therapeutic or diagnostic purposes (Ahmad et al., 2018). Chitosan (CS) is a widely used gene delivery nanocarrier in recent years. It is a product of chitin deacetylation and has the advantages of being nontoxic, abundant, biocompatible and biodegradable. The presence of hydrogen bonds between CS molecules makes it insoluble in organic solvents and water, and the resulting drug delivery system is readily ingested by the Retieuloendothelial System (RES) (Ait Bachir et al., 2018). Polyethylene glycol Polyethylene glycol (PEG) is a branched or straight-chain polymer based on the structure of -CH2CH2O- and the solubility of PEG-modified CS is increased in aqueous and organic solutions (Fang et al., 2018). CS modified with PEG has increased solubility in aqueous and organic solutions, with methoxy polyethylene glycol (mPEG) derivatives being the most widely used (D’souza & Shegokar, 2016). The PEG-modification of CS results in reduced efflux, enhanced permeation and retention (EPR) (Javid et al., 2020). So the drug has been modified with PEG to reduce efflux and enhance permeation and retention. Paclitaxel (PTX) is a natural anticancer drug isolated from the bark and needles of the redbud plant. It is a cell cycle blocker that stabilizes microtubules and leads to cell growth inhibition and apoptosis (Ghofrani et al., 2019). However, PTX has the disadvantages of poor targeting and toxic side effects when used in systemic chemotherapy (Lin et al., 2020). B cell specific MLV integration site-1 (*Bmi-1*) is a member of the polycomb family of transcriptional regulators, which mediate a signaling system involved in the self-renewal of many CSCs (Zhang et al., 2016). The study stressed that it is associated with lymphatic metastasis and drug resistance in laryngeal cancer (Yu et al., 2015). Our group has shown that the proto-oncogene *Bmi-1 is a* key locus of self-renewal in laryngeal CSCs, and that targeted down-regulation of *Bmi-1* can effectively inhibit the proliferation and differentiation of laryngeal CSCs, making them more sensitive to the chemotherapeutic agent PTX (Wei et al., 2018). However, in humans, the unencapsulated *Bmi-1RNAi* nucleotide fragment is susceptible to degradation by nuclease attack in body fluids, and lacks targeting to tumor tissues and cannot be effectively introduced into target cells (Liu et al., 2015). Glycine aspartic acid (RGD) peptides are short peptides containing the arginine-glycine-aspartic acid (ARG-GLY-ASP) sequence, which are recognition sites for the interaction of integrins αv (ITG αv) and ITG β3 with their ligand proteins. RGD helps anchor cells in both directions to the extracellular matrix (ECM). Among them, ITG αv and ITG β3 are highly expressed on the surface of laryngeal cancer cells (Jin et al., 2020). It was noted that cyclic RGD (cRGD) had higher receptor binding specificity than linear RGD peptides (Gajbhiye et al., 2019). The cRGD peptide specifically binds to ITGαvβ3 on the surface of laryngeal CSCs. It competes with the extracellular matrix αvβ3 receptor to inhibit the exogenous RGD peptide and reduce the adhesion, metastasis and invasion of laryngeal cancer cells, while activating the apoptosis-related gene Caspase-3 to induce apoptosis in tumor cells (Shao et al., 2015). In this study, we proposed to synthesize *Bmi-1RNAi* recombinant plasmids, then construct cRGD peptide-modified chitosan-polyethylene glycol slow-release nanoparticles (cRGD/CS-PEG-*Bmi-1RNAi*-PTX) co-loaded with PTX and *Bmi-1RNAi,* and investigate their morphology, particle size, potential, cytotoxicity, gene protection and other physicochemical properties. In vitro transfection of Hep-2 cells and laryngeal CSCs. In vivo inhibition of laryngeal cancer in mice by tail vein administration with combination of cRGD peptide, PTX and *Bmi-1RNAi.* Overlapping mechanism of multi-targeted killing of laryngeal CSCs by cRGD peptide, PTX and *Bmi-1RNAi* were investigated.

## Materials and methods

### Cell culture and sorting

Hep-2 cells (BD Biosciences, Beijing, China) were cultured in RPMI1640 culture medium (Hyclone, USA) containing 10% calf serum (FBS, Gibco, USA) and 100 U/mL of penicillin in a 5% CO2 incubator at 37 °C and passaged every 3-4 days. After sorting by flow cytometry (BD Biosciences, Hercules, CA, USA), CD133^+^ and CD133^−^ were cultured in fresh RPMI1640 complete medium, respectively. All cell lines were checked for mycoplasma every three months and confirmed free of infection prior to use.

### Synthesis and characterization of mPEG-CS-cRGD/*Bmi-1RNAi*-PTX

#### Preparation of *pGenesil-Bmi-1RNAi* plasmids

The siRNA targets were designed according to the general principles of shRNA design. According to the primer oligonucleotide sequence 5 -AAACCTAATACTTTCCAGATTGATTT GGATCCAAATCAATCTGGAAAGTATTAGG-3′(NM-005180, nt1061-1081), double stranded oligonucleotide RNA with hairpin structure was designed and synthesized. Ligation of the interfering fragment to the expression vector, transformation and plasmid sequence analysis to determine the correct synthesis of the new plasmid *pgenesil-Bmi-1.* After transformation treatment, the down-regulation of *h-BMI1* interference on 293 T cells (Shanghai Hanheng Biotechnology Co., Ltd., Shanghai, China) was analyzed using real-time fluorescence quantitative PCR (qRT-PCR).

### Synthesis and characterization of mPEG-CS

An amount of CS (120,000 Da, deacetylation >99%) (Guangzhou Carbon Water Technology Co., Ltd., Guangzhou, China) was dissolved in 1% acetic acid solution to make a master batch of 10 mg/mL. 30 mg MPEG-COOH (2000 Da) (Guangzhou Carbon Water Technology Co., Ltd., Guangzhou, China) was dissolved in 3 mL of 1% acetic acid solution and 2.5 times the molar amount of mPEG-COOH was added to 1-ethyl-3-(3-dimethylaminopropyl)-carbodiimide (EDC) and N-hydroxysuccinimide (NHS) (Shanghai Aladdin Reagent Co). The CS master batch was gradually added dropwise to the activation solution and the pH was adjusted to 5.0 with sodium hydroxide (NaOH) (Sinopharm Chemical Reagent Co., Shanghai, China), then the reaction was stirred at room temperature for 48 h. After the reaction was completed, the solution was transferred to a molecular weight cut off (MWCO) of 8000–14000 Da. After the reaction, the solution was transferred to a dialysis bag (Spectra/Por®) with a MWCO of 8000–14000 Da and dialyzed in pure water for 48 h. Structural characterization was performed by 1H-NMR.

### Preparation of mPEG-CS-cRGD

Dispense 10 mg/mL mPEG-CS mother liquor. 5 mg of cRGD was dissolved in 10 mL 1% acetic acid solution, and EDC and NHS with twice the molar amount of cRGD were added for activation for 2h. 2 mL of CS mother liquor was gradually added into the activation solution by drops, and the pH was adjusted to 5.0 with NaOH solution. Then, after 24h stirring reaction at room temperature, it was placed in pure water for 48h dialysis and freeze-dried. At the end of the reaction, the solution was transferred to a dialysis bag with a MWCO of 3500 Da. After the reaction was completed, the solution was transferred to a dialysis bag (Spectra/Por®) with a MWCO of 3500 Da, dialyzed in pure water for 48 h and then freeze-dried.

### Preparation of mPEG-CS-cRGD/PTX nanoparticles

1-Ethyl-3-(3-dimethylaminopropyl)-carbonized diimide (EDC) and N-Hydroxysuccinimide (NHS) were added into 30 mg mPEG-COOH (2000 Da), activated for 30 min, and then CS mother liquor (10 mg/mL) was added. mPEG-CS was synthesized by freeze-drying after 48h reaction and 48h dialysis at pH 5.0. 5 mg of cRGD was added into EDC and NHS with twice the molar amount of cRGD. After activation for 2h, it was added into 10 mg/mL mPEG-CS mother liquor. 2 mL of CS mother liquor was gradually added into the activation solution by drops. At pH 5.0, after reaction for 24h and dialysis for 48h, freeze drying was carried out to synthesize mPEG-CS-cRGD/PTX

PTX (Shanghai Yuanye Biotechnology Co., Ltd., Shanghai, China) loaded mPEG-CS nanoparticles were prepared by ultrasonic method. Typically, PTX was dissolved in acetone to a concentration of 2.5 mg/mL. 1 mL of this solution was taken and 10 mL of mPEG-CS-cRGD solution (2 mg/mL in distilled water) was added in an ice bath (pulsed on for 2 s and off for 2 s) for 30 min under the action of ultrasound at 750 W. The mixture was then dialyzed with pure water for 48 h (molecular weight of 30 kDa). Unloaded PTX was removed by centrifugation at 4000 rpm for 10 min. The supernatant was freeze-dried to obtain PTX-loaded mPEG-CS-cRGD nanoparticles.

### Preparation and characterization of nanoparticles loaded with Bmi-1 RNAi gene

A simple complexation method was used to prepare mPEG-CS-cRGD/Bmi-1RNAi-PTX nanoparticle complexes. Firstly, mPEG-CS-cRGD/PTX was dissolved in acetate buffer at a final concentration of 2.5 mg/mL and filtered through a 0.22 μm micro porous membrane, and 20 μg of *Bmi-1RNAi* in ribonuclease-free water was used for pellet formulation. The mPEG-CS-cRGD/PTX NPs with different CS: mPEG molar ratios (Table 1) were stirred with 20 μg of Bmi-1RNAi at room temperature under constant magnetic stirring for 1h.

**Table 1.** Different ratio of CS and mPEG with 20 μg *Bmi-1 RNAi.*

*Bmi-1RNAi(μg)*	NPs 1:60(μg)	NPs 1:90(μg)	NPs 1:180(μg)
20	2.16	2.27	2.61
	21.59	22.57	26.13
	32.38	33.85	39.20
	64.77	67.71	78.39

### Particle size, potential determination and morphological observations

Prepared solutions of nanoparticle complexes with different dosing ratios (CS:mPEG) were added to the Zetasizer Nano ZS laser particle sizer cuvette Then the nanoparticle size was determined by DLS and the potential was measured by ELS. The prepared nanoparticles were diluted a certain number of times and added dropwise to the copper mesh, which was allowed to dry and then placed onto a transmission electron microscope for observation and photography.

### Calculation of EE and DLE

Two equal parts of the prepared solution loaded with PTX nanoparticles without precipitation were taken. One part was centrifuged and the supernatant was taken. The concentration of PTX was measured by UV light (maximum absorption at 231 nm wavelength). After the precipitation was freeze-dried by centrifugation in the other part, PTX in the carrier was extracted by methanol with appropriate amount of powder, and then measured and calculated by UV light (the precipitate was the free PTX not wrapped by nanoparticles, and the supernatant was the PTX coated with nanoparticles). The encapsulation efficiency (EE) and drug loading efficiency (DLE) were then calculated according to the following equations. Where We is the amount of drug encapsulated in the carrier, Wo is the amount of free drug not encapsulated, and Wm is the total weight of the carrier.

$$EE=\frac{W_{e}}{W_{e}+W_{o}}*100\%$$

$$\mathrm{DLE}=\frac{W_{e}}{W_{m}}*100\%$$

### mPEG-CS vector wrapping DNA capacity

Nanoparticles of different vector gene ratios (w/w) (0:1, 0.25:1, 0.5:1, 1:1, 2:1, 3:1, 4:1, 6:1, 8:1, 10:1, 12:1, 16:1, 24:1, 32:1) were prepared by using mPEG-CS as the carrier. The nanoparticles were mixed with the loading buffer and added to the loading wells of the agarose gel. The gel was abducted and photographed under UV light.

### Resistance of mPEG-CS NPs to nuclease degradation

Nanoparticles of different vector gene ratios (w/w) were prepared. DNase I (1 U/μL) was added to the reaction buffer and incubated for 30 min at 37 °C. After incubation, EDTA (50 Mm) was added. DNase I was inactivated by heating at 37 °C for 20 min to terminate the enzymatic reaction, followed by the addition of 5 μg/μL of heparin and left for 60 min at 37 °C for gel electrophoresis blocking experiments.

### Evaluation of the gene silencing effect of gene loaded nanoparticles

Hep-2 cells were passaged and inoculated in 6-well cell culture plates with a cell number of 1x10^5^, and transfection was started when cell fusion reached approximately 60%. Blank control group, a naked gene group, mPEG-CS-cRGD/*Bmi-1RNA*i-PTX group and liposome group were set up. After transfection, the cells were expected continue to be cultured for 24 h, then collected. qRT-PCR was used to detect the expression of *Bmi-1* in the cells. We statistically analyzed with the expression before silencing to observe the treatment effect.

### Evaluation of the effect of nanoparticle transfection

Hep-2 cells were transfected with plasmid pEGFP-C3, encoding enhanced green fluorescent protein, and the transfection was observed in vitro by inverted fluorescence microscopy. Hep-2 was inoculated and cultured in 24-well cell culture plates such that cell fusion reached 70-80% in each well at the time of administration. The mPEG-CS-RGD/pEGFP-C3 nanosuspension was added to the wells after filtration and de-sterilization. The naked gene group, liposome group, mPEG-CS-cRGD group and mPEG-CS-cRGD/PTX group were established. After 6h of administration, the culture medium was replaced to complete medium and continued for 24 h. The green fluorescent protein (GFP) in the cells was observed by inverted fluorescence microscopy. The expression of green fluorescent protein (GFP) was observed by inverted fluorescence microscopy.

### Effect of mPEG-CS-cRGD/Bmi-1RNAi-PTX on the proliferation of Hep-2 cells

Hep-2 cells at the logarithmic growth stage were counted on cell counting plates and spread in 96-well cell culture plates, corresponding to 1 × 10^3^ cells per well (Wang et al., 2020). Each well was incubated with PTX (10, 100, 200, 500, 1000 and 5000 ng/mL) to a final volume of 100 uL per well. Six replicate wells were set up for negative control (no drug treatment) and blank control (same volume of culture medium added). After each well was incubated for 24 h and 48 h under the same conditions, the culture medium was discarded and 10 uL of cck-8 solution was in addition to each well and incubated for 3 h. The absorbance (A value) of each well was measured at 450 nm on an ELISA. Depending on the A value, the cell viability after treatment with the vector was calculated, and the IC50 of PTX to Hep-2 cells was obtained. Cck-8 cell proliferation-toxicity assay was utilized to detect the respective proliferation inhibition rates of PTX, mPEG-CS/PTX, mPEG-CS-cRGD/PTX and mPEG-CS-cRGD/*Bmi-1RNAi*-PTX on Hep-2 cells. Hep-2 cells at the logarithmic growth stage were given equal amounts of PTX, mPEG-CS/PTX, mPEG-CS-cRGD/PTX and mPEG-CS-cRGD/*Bmi-1RNAi*-PTX in 96-well cell culture plates (their final concentration corresponded to IC50 of PTX). Six repeat wells were set up in each group, and the negative control group and blank control group were also develop. The cell inhibition rate among each group was calculated after incubation for 24 h and 48 h. *Cell Viability* was calculated as following.

$$\mathrm{Cell}\;\mathrm{Viability}\left(\%\right)\;=\;\left(A-A_{0}\right)/(A_{\mathrm{control}}-A_{0})x\;100\%$$

Where A represents the absorbance of the cell group treated with the complex. A_0_ represents the absorbance of the blank medium solution. A_control_ represents the absorbance of the cell group untreated with the preparation.

### Flow cytometry sorting of CD133+ laryngeal CSCs

Hep-2 cells at the logarithmic growth stage were washed twice in pre-chilled PBS solution, digested by adding 0.25% trypsin, terminated with complete medium and washed and resuspended in DPBS buffer containing 5 mM EDTA, 25 mM HEPES (pH 7.0) and 1% BSA. Adding 200 uL of buffer with resuspended, then FITC-labelled CD133 antibody (Bioscience) was added at a ratio of 1:100 of 2uL, then incubated for 30 min at 4 °C. After that taking to wash, filtered, then the proportion of positive cells was detected by flow cytometry (Qiu et al., 2016). The percentage of positive cells was detected by flow cytometry. CD133^+^ and CD133^-^ cells were successfully sorted by flow cytometry and incubated in serum-free medium (SFM) with B-27^TM^ (2%v, Gibco), basic fibroblast growth factor (bFGF, 20 ng/mL, Gibco), epidermal growth factor (EGF, 20 ng/mL, MACGENE, Shanghai, Beijing), and insulin (5 ug/mL, MACGENE, Beijing, China). The sorted cells were cultured in an incubator at 5% CO_2_ 37 °C, and observed every 2 days for a total of 9 days (Zhou et al., 2007).

### Enrichment of laryngeal cancer stem cells with the chemotherapeutic drug PTX

Hep-2 cells at the logarithmic growth stage were added to a 6-well cell culture plates at an inoculum density of 2 × 10^5^ cells. After the cells were plastered, they were co-cultured with PTX for 48 h (the final concentration of PTX was the IC_50_ of Hep-2 cells of 90.11 ng/mL). They were replaced with fresh medium and continued to be cultured for 24 h. The above steps were replicated 3 times, and the resulting cells were chemotherapy-enriched laryngeal CSCs (Steinbichler et al., 2018).

### Colony formation test

Five groups of control (PBS), Fee-PTX, mPEG-CS/PTX, mPEG-CS-cRGD/PTX and mPEG-CS-cRGD/*Bmi-1RNAi*-PTX were set up. They are applied to Hep-2 cells in the logarithmic phase of apposed growth (the final concentration of PTX was the IC_50_ of Hep-2 cells of 90.11 ng/mL) for 48 h. 3 replicate values were established for each group, and the cells were grown in 6-well cell culture plates at 5x10^2^ cells per well, with fluid changes every 2 days. Cells were incubated for 2 weeks in a cell culture incubator at 5% CO_2_ and 37 °C, then washed twice with PBS and fixed in 4% paraformaldehyde, followed by staining with crystalline violet solution for 30 min. Finally photographed and colony formation were counted (Jiang et al., 2020).

### Cell scratch test

Hep-2 cells in the logarithmic phase of apposed growth were inoculated with 2 × 10^5^ cells in 6-well cell culture plates, and the cells were fused to approximately 80% of the total number of cells (Huang et al., 2020). After respectively treatment with Free PTX, mPEG-CS/PTX, mPEG-CS-cRGD/PTX and mPEG-CS-cRGD/*Bmi-1RNAi*-PTX (the final concentration of PTX was the IC_50_ of Hep-2 cells of 90.11 ng/mL), the surface of the plate was scratched with a 200 μL sterile pipette tip to form a trabecular surface. After co-culture for 0 h, 24 h and 48 h, respectively, images of the scratched trabeculae were taken under an inverted phase contrast microscope and 20x magnification. Finally, the trabeculae area was calculated using ImageJ software.

### Annexin V-FITC/PI double staining method

Hep-2 cells at logarithmic growth stage were inoculated in 12-well cell culture plates with 5x10^4^ cells per well (Wang et al., 2020). After cell fusion reached approximately 80%, the cells were incubated with equal amounts of mPEG-CS, mPEG-CS/PTX, mPEG-CS-cRGD/PTX and mPEG-CS-cRGD/*Bmi-1RNAi*-PTX for 24 h (the final concentration of PTX was the IC_50_ of Hep-2 cells of 90.11 ng/mL). A negative control group was established with 3 replicate wells for each group. Each group was created with 3 replicate wells. Cells were digested with EDTA-free trypsin, collected by centrifugation and stained with Annexin V-FITC/PI according to the kit protocol. After staining they were examined on a flow cytometer immediately and the percentage of apoptosis cells was analyzed using Flow Jo V10 software.

### qRT-PCR

Hep-2 cells at the logarithmic growth stage were collected after PTX chemotherapy enrichment and cultured in 6-well cell culture plates according to 2 × 10^4^ cells. After cell fusion reached approximately 80%, the cells were treated with pre-configured Free PTX, mPEG-CS/PTX, mPEG-CS-cRGD/PTX and mPEG-CS-cRGD/*Bmi-1RNAi*-PTX for 48 h (the final concentration of PTX was the IC_50_ of Hep-2 cells of 90.11 ng/mL). Total RNA was extracted from the treated Hep-2 cells using Trizol kit (USA), and reverse transcription was carried out using the PrimeScript RT Reagent Kit (Japan). Primers (Table 2) were synthesized in advance (Shanghai PrimeTech Biotechnology Co., Ltd., Shanghai, China). The concentration of RNA was measured by photometer and samples with an A260/A280 ratio of 1.8–2.0 were selected for the next qRT-PCR experiment. qRT-PCR amplification was completed by SYBR GREEN quantitative PCR reagent at 94 °C for 15 s, 55 °C for 30 s and 72 °C for 30 s, for a total of 40 cycles. Because glyceraldehyde-3-phosphate dehydrogenase (GAPDH) was stable in all experimental groups, it served as an internal reference and was quantified using the relative. The changes were calculated using the relative quantification method (2-ΔΔCt method). The results were automatically recorded and generated by the QuantStudio™ Real-Time PCR Software computer system.

**Table 2.** Primers used in real-time PCR.

	Forward sequence	Reverse sequence
*Gapdh*	GTCTCCTCTGACTTCAACAGCG	ACCACCCTGTTGCTGTAGCCAA
*Caspase-3*	GGAAGCGAATCAATGGACTCTGG	GCATCGACATCTGTACCAGACC
*Bcl-2*	ATCGCCCTGTGGATGACTGAGT	GCCAGGAGAAATCAAACAGAGGC
*Bax*	TCAGGATGCGTCCACCAAGAAG	TGTGTCCACGGCGGCAATCATC
*Bmi-1*	GGTACTTCATTGATGCCACAACC	CTGGTCTTGTGAACTTGGACATC
*Oct-4*	CCTGAAGCAGAAGAGGATCACC	AAAGCGGCAGATGGTCGTTTGG
*Sox-2*	GCTACAGCATGATGCAGGACCA	TCTGCGAGCTGGTCATGGAGTT
*Nanog*	CTCCAACATCCTGAACCTCAGC	CGTCACACCATTGCTATTCTTCG
*C-myc*	CCTGGTGCTCCATGAGGAGAC	CAGACTCTGACCTTTTGCCAGG
*ITG av*	AGGAGAAGGTGCCTACGAAGCT	GCACAGGAAAGTCTTGCTAAGGC
*ITG β3*	CATGGATTCCAGCAATGTCCTCC	TTGAGGCAGGTGGCATTGAAGG

### Western blotting

After Hep-2 cells with cell and tissue were treated with the method used for qRT-PCR as described above, total protein was extracted with RIPA solution, 50-80 μg of protein was separated by 10% polyacrylamide gel electrophoresis, and transferred to polyvinylidene fluoride membranes (PVDF membranes, USA). Tumor stem cell-associated markers CD133 (Rabbit monoclonal (EPR16508) to CD133, abcam, 1:2000), Bmi-1 (Bmi1 (DC9) Mouse mAb, Cell signaling technology, 1:1000), C-myc (C-myc Mouse mAb, Proteintech, 1:5000), Nanog (Nanog Mouse mAb, Proteintech, 1:5000), Oct-4 (Oct-4 Mouse mAb, Proteintech, 1:5000), Sox-2 (Sox-2 Mouse mAb, Proteintech, 1:5000), apoptosis-related genes B-cell lymphoma-2 (Bcl-2) (Rabbit monoclonal (E87) to Caspase-3, abcam, 1:5000), Caspase-3 (Rabbit monoclonal (EPR18297) to Caspase-3, abcam, 1:5000), Bcl-2 associated X (Bax) (Rabbit monoclonal (EPR18283) to Bax, abcam, 1:2000) and GAPDH (GAPDH Mouse mAb, abcam, 1:5000) were incubated overnight. The next day, after incubation with horseradish peroxidase-labelled secondary antibody for 1h, the membrane was washed with hydrochloric acid buffer containing earth temperature (Tris-buffered Saline Tween-20, TBST). Finally, the membrane was blotted with enhanced chemiluminescence solution (ECL) for protein detection. The results were recorded by a Tennant (Tanon 3500, Shanghai, China) series fully automated gel image analysis system. Protein bands were quantified using Image Lab 3.0 software (Bio-RAD, Inc., Hercules, CA, USA).

### In vivo tests

BALB/c nude immunodeficiency mice (Beijing, China, license no. SCXK (Beijing) 2019-0010), all mice (30 males, all 5-6 weeks old, weighing 18-21 g) were feed SPF-grade chow and purified water, and approved by the Laboratory Animal Ethics Committee of Laboratory animal center of Lanzhou University (LZUSYDWZX-2021-034) according to national animal welfare requirements, and room temperature was maintained at 20-22 °C. Mice were randomly divided into five groups Free PTX, mPEG-CS/PTX, mPEG-CS-cRGD/PTX and mPEG-CS-cRGD/*Bmi-1RNAi*-PTX, with six mice in each group. Hep-2 cells of laryngeal carcinoma at logarithmic growth stage were taken, prepared into cell suspension and subcutaneously injected into the back of the neck with a dose of 5 × 10^6^/mL each mice and 100 μL of each mouse. Tumor size needed to be measured and weighed starting the day after inoculation, and the health status of nude mice was observed daily. Tumor growth was calculated as V = (aXb^2^)/2 (a: long diameter, mm. b: short diameter, mm) (Yan et al., 2020). PTX, mPEG-CS/PTX, mPEG-CS-cRGD/PTX and mPEG-CS-cRGD/*Bmi-1RNAi*-PTX each mice (PTX 5 mg/kg, *Bmi-1RNAi* 10 ug of each mice) were injected into the tail vein of BALB/c nude mice on the 7th day after subcutaneous injection of Hep-2 cells, the tumor volume reached approximately 100 mm^3^ (Hyun et al., 2021). A total of 6 doses were administered. The tumor size and tumor volume were measured before each dose, and the tumor growth curve was plotted. When the tumors in the control group reached approximately 300 mm^3^, the mice were executed and tumors were peeled off to compare the size of transplanted tumors and the weight change of mice in the five groups.

### Tumor tissue stained with hematoxylin and eosin (H&E staining)

Mice were addressed in the above counterparts for 19 days. Then the heart, kidney, liver and tumor tissues were taken for histological evaluation using hematoxylin and eosin staining. Tissues were fixed in 4% paraformaldehyde, paraffin sections were made, stained with hematoxylin and eosin according to literature tips and standard procedures, then photographed under a Lycra orthomosaic microscope at 200×. Finally, the proportion of positive staining was quantified using ImageJ software (Jia et al., 2017).

### Immunohistochemistry

Tissues were fixed in 4% paraformaldehyde, dehydrated, permeabilised and embedded, then cut into 5 um sections, incubated overnight with the primary antibody used for the experiments. Combined with the analogous horseradish peroxidase, finally stained, sealed, photographed under 200x in a positive microscope (Leica) and the positive staining ratio quantified using ImageJ software (Hua et al., 2018; Huang et al., 2020).

### Data analysis

All data are expressed as mean ± standard deviation. All data were statistically analyzed using SPSS 26.0 software, and images were produced by GraphPad Prism 8.0, Photoshop and ImageJ software. Statistical differences were analyzed using one-way analysis of variance (ANOVA), with p-values <0.05 considered significant, and all statistically different data are uniformly denoted by *.

## Results

### Evaluation of the interference effect of BMI-1-shRNA recombinant plasmids

The h-BMI1-shRNA was developed, synthesized and linked with the pGenesil-1 plasmid vector to construct a recombinant plasmid. The sequencing results showed that all three designed h-BMI1 shRNAs were inserted correctly, and the sequences measured were consistent with the target sequences (Supplementary Figure 1 ai ∼ aiii, b). The solubility curves of GAPDH and h-BMI1-shRNA were good, all performed effective amplification (Supplementary Figure 1ci-ciiii). All three h-BMI1-shRNAs were down-regulated in 293 T cells, and the relative expression of *Bmi-1* after down-regulation was respectively to 0.118 ± 0.003, 0.124 ± 0.017 and 0.172 ± 0.007 (Supplementary Figure 1d). h-BMI1-shRNA1, which had the best interference effect, was chosen for subsequent experiments. shRNA1 plasmid with nanocarriers was selected for subsequent experiments to construct nano-slow-release particles.

**Figure 1.**
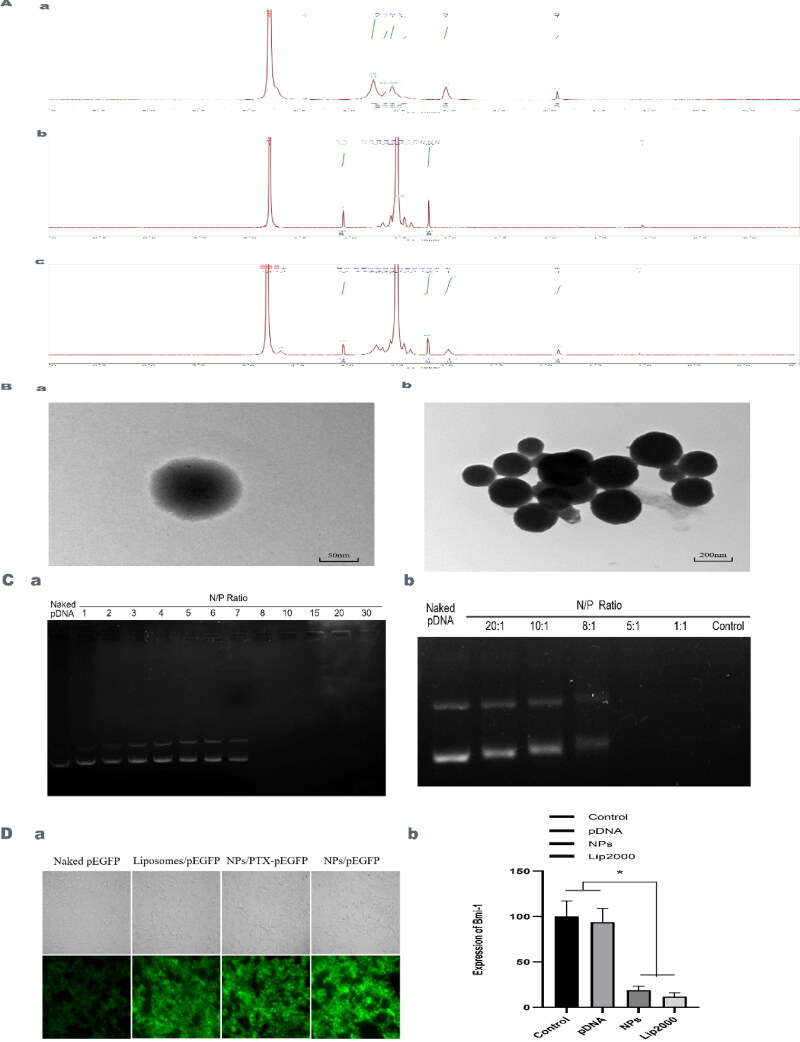
**A.** Synthesis and Characterization of mPEG-CS-cRGD/*Bmi-1RNAi*-PTX. ^1^H-NMR of CS (a), mPEG-COOH (b) and mPEG-CS (c). **B.** TEM showed that the nanocomposite was approximately spherical with a particle size of about 200 nm (a: 50 nm, b: 200 nm). **C.** The mPEG-CS carrier has the ability to encapsulate DNA and resisted nuclease degradation. a When N/P ratio was 8, pDNA was completely wrapped. b When N/P ratio was less than 5, the DNA bands disappeared. **D** Package with DNA ability and anti-nucleic enzyme degradation ability of mPEG-CS. a mPEG-CS-cRGD NPs have the better transfection effect to naked DNA. b mPEG-CS-cRGD/*Bmi-1RNAi*-PTX NPs silenced *Bmi-1* gene expression. We use one-way ANOVA exposure, control and pDNA vs. other two groups, f(3)=36.786, *p* < 0.001.

### Synthesis and characterization of mPEG-CS-cRGD/Bmi-1RNAi-PTX

Characterization of the synthesized mPEG-CS-cRGD/*Bmi-1RNAi*-PTX showed that the peak at 3.40-3.76 ppm was the H peak on the chitosan glucosamine ring, the peak at 3.03 ppm was the H peak at position 2 on the chitosan glucosamine ring, and the peak at 1.92 ppm was the H peak of the unabrogated acetyl terminal methyl group on chitosan (Figure 1Aa). 4.07 ppm is the methylene H peak on mPEG attached to the carboxyl group. 3.21 ppm is the methoxy H peak on mPEG (Figure 1Ab). 4.03 ppm is the methylene H peak on PEG. 3.19 ppm is the H peak on mPEG methoxy. 2.98 ppm is the H peak at the 2 position on the chitosan glucosamine ring. 3.64 ppm is the methylene on the amide bond attached to the CS of mPEG methyl group, and 1.0 ppm is the H peak of the unremoved acetyl terminal methyl group on the chitosan (Figure 1Ac). This shows that mPEG was successfully attached to CS. In the 1H-NMR of mPEG-CS, the characteristic peaks of both mPEG and CS were present. We have different CS:mPEG feeding ratios, when the CS:mPEG feeding ratio was 1:180, the particle size of the nanocomplex at this time was 182.3 ± 30.6 nm, the zeta potential was 10.14 ± 0.52 MV, the DLE was 4.24% and the EE was 84.84%. Comparing to the CS:mPEG feeding ratios of 1:90 and 1:60, the zeta potential of 1:180 was higher, while the DLE was more or less the same, the EE was lower than for 1:60 and more or less the same as for 1:90 (Table 3). TEM showed that the nanoparticle complexes were nearly spherical and more uniformly distributed, with particle sizes around 200 nm (Figure 1B).

**Table 3.** The diameter, zeta potential (mV), DLE and EE of NPs with different ratio of CS with mPEG.

CS: mPEG unit(mg:mg)	d(nm)	zeta potential(mV)	DLE	EE
1:60	398.5 ± 47.9	2.9 ± 0.71	4.57%	91.42%
1:90	244.9 ± 38.4	5.29 ± 0.44	4.09%	81.88%
1:180	182.3 ± 30.6	10.14 ± 0.52	4.24%	84.84%

### mPEG-CS vector encapsulation of Bmi-1 and resistanced to nuclease degradation

Nps with different w/w ratios were prepared using mPEG-CS as the carrier, added to the sample wells of agarose gels, electrophoresis in an electric field, then the gels were removed and photographed under UV light. When N/*P* = 8, pDNA no longer spilled out of the sample wells, indicating that pDNA was completely wrapped by the vector (Figure 1Ca). When the N/P was lower than 5, the Bmi-1 bands disappeared, indicating that the vector could not protect the loaded nucleic acid at all, and the nucleic acid was degraded by nuclease (Figure 1Cb). So, 15:1 N/P mPEG-CS-cRGD/*Bmi-1RNAi*-PTX nanoparticle complex was prepared.

### Gene silencing effect of mPEG-CS-cRGD loaded nanoparticles

After transfection of cells utilizing vectors loaded with fluorescent protein genes, the intensity of fluorescence was observed, and the higher intensity indicated a better transfection effect. mPEG-CS-cRGD NPs group showed a stronger fluorescence intensity compared with liposome transfection, simple nanoparticles and nanoparticles loaded with PTX, indicating a good transfection effect (Figure 1Da). Hep-2 cells were co-cultured with mPEG-CS-cRGD/*Bmi-1RNAi*-PTX Nps when the degree of fusion reached about 60%, and the expression of *Bmi-1RNAi* was observed by qRT-PCR. The results showed that, comparing with blank control, bare gene and liposome transfection, mPEG-CS-cRGD/*Bmi-1RNAi*-PTX Nps significantly silenced *Bmi-1* gene expression by 0.172 ± 0.007 (*p* < 0.001) (Figure 1Db).

### Effect of mPEG-CS-cRGD/Bmi-1RNAi-PTX on the proliferative capacity of Hep-2 cells

**Figure 2.**
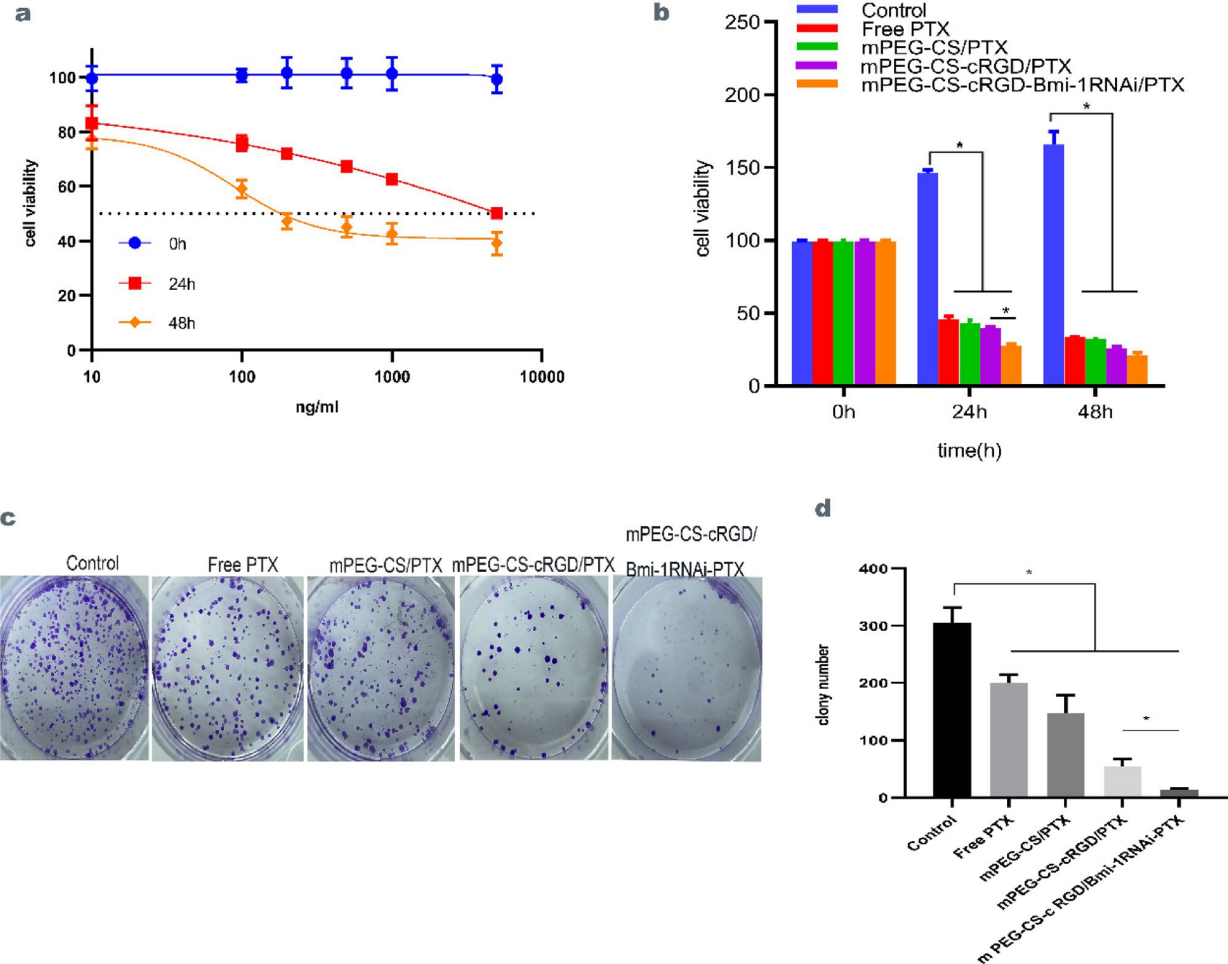
mPEG-CS-cRGD/*Bmi-1RNAi*-PTX inhibited the proliferation of Hep-2 cells in vitro. a The IC50 of PTX on Hep-2 cells was calculated about 90.11 ng/mL. b Comparing to control group, the cytotoxicity of each group was significantly raise, with time-dependent, we use one-way ANOVA-exposure, control vs. other four groups, 24h: f(4)=2438.14, *p* < 0.001. 48h: f(4)=633.84, *p* < 0.001. c Comparing with the control group, the cells incubated with mPEG-CS-cRGD/*Bmi-1RNAi*-PTX have the least cluster. d We use one-way ANOVA exposure, control vs. other four groups, f(4)=94.04, *p* < 0.001.

The effect of mPEG-CS-cRGD/Bmi-1RNAi-PTX on the proliferation of Hep-2 cells was observed by the cck-8 proliferation-toxicity assay. After respectively incubation of different concentrations of PTX with Hep-2 cells for 24 h and 48 h, the IC_50_ of PTX on Hep-2 cells was calculated to be 90.11 ng/mL (Figure 2a). With PTX, mPEG-CS/PTX, mPEG-CS-cRGD/PTX and mPEG-CS-cRGD/*Bmi-1RNAi*-PTX (corresponding to a PTX concentration of 90.11 ng/mL) respectively acted on Hep-2, the results showed that the cytotoxicity of each group increased significantly with the prolongation of the action time, with the largest cytotoxicity corresponding to 48 h, reflecting a time-dependent (*p* < 0.001) (Figure 2b). Being Compared to the untreated group, the number of Hep-2 cell colonies formed after the action of mPEG-CS-cRGD/*Bmi-1RNAi*-PTX was the lowest and the inhibition was significant (*p* < 0.001) (Figure 2c ∼ d). The order of the strong and weak toxic effects of the four groups on cells was: mPEG-CS-cRGD/*Bmi-1RNAi*-PTX > mPEG-CS-cRGD/PTX > mPEG-CS/PTX > Free-PTX. It showed that mPEG-CS-cRGD/*Bmi-1RNAi*-PTX could inhibit Hep-2 cell proliferation and decrease their colony-forming ability.

### Effect of mPEG-CS-cRGD/Bmi-1RNAi-PTX on the expression of CD133, C-myc, Nanog, Oct-4 and Sox-2 in Hep-2 cells

**Figure 3.**
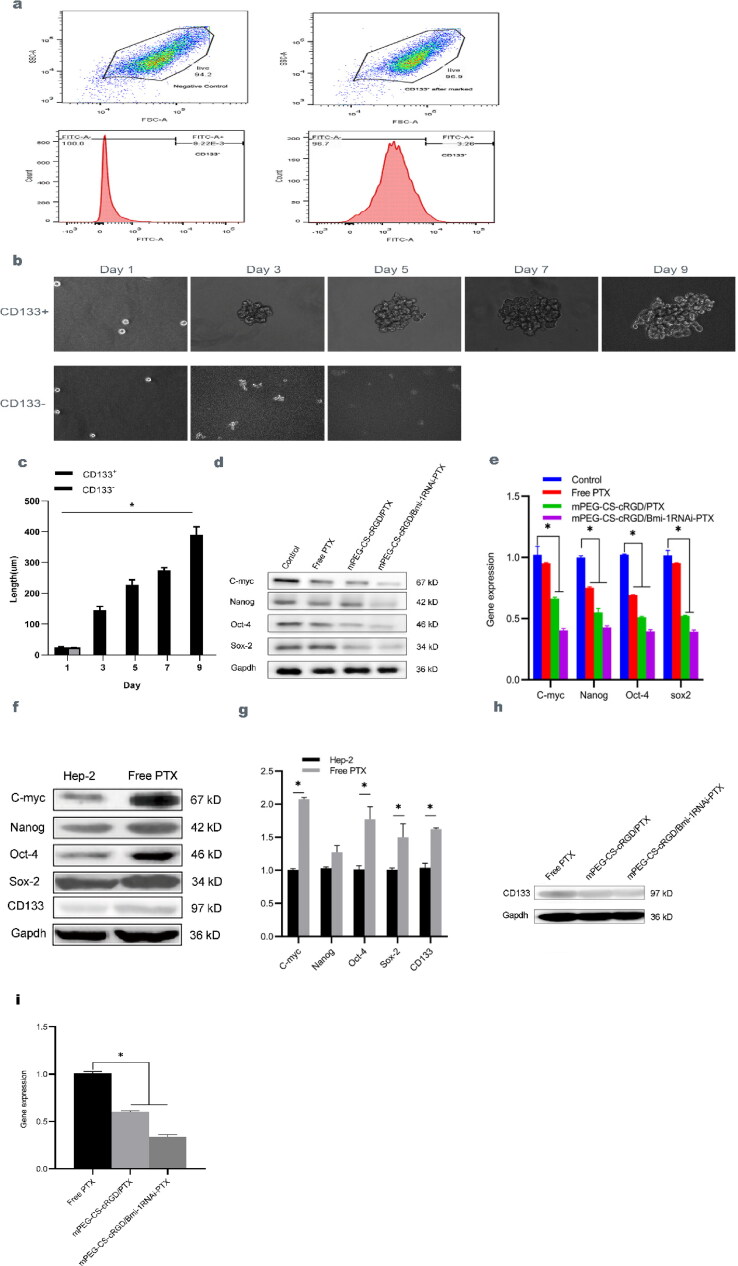
a The expression of CD133 ratio in Hep-2 cells detected by Flow cytometry. b CD133^+^ and CD133^-^ cells were suspended under an inverted microscope after sorting, the cell pellet diameter of CD133^-^ increased with the increase of days. c The diameters of cell spheres were measured and pairwise comparisons were made on days 1, 3, 5, 7 and 9. We use one-way ANOVA-exposure, f(4)=236.631, *p* < 0.01. d The protein level of C-myc, Nanog, Oct-4 and Sox-2 in Hep-2 cells teated with mPEG-CS-cRGD/*Bmi-1RNAi*-PTX were reduced. e qRT-PCR results have showed the consistent with Western blot, we use one-way ANOVA exposure, control vs. other three groups, C-myc: f(3)=185.08, *p* < 0.001. Nanog: f(3)=556.31, *p* < 0.001. Oct-4: f(3)=3691.10, *p* < 0.001. Sox-2: f(3)=612.14, *p* < 0.001. f The protein and expression levels of CD133, C-myc, Nanog, Oct-4 and Sox-2 of Western blot. g qRT-PCR show the consistent with Western blot, we use t-test exposure, Hep-2 vs. Free PTX, C-myc: t(4)=-59.168, *p* < 0.001. Nanog: there were statistical difference. Oct-4: t(4)=-6.626, *p* < 0.05. Sox-2: t(4)=-4.12, *p* < 0.05. CD133: t(4)=-14.67, *p* < 0.001. h The protein level of CD133 was detected by western blotting after these drug-resistant cells treated with mPEG-CS-cRGD/PTX and mPEG-CS-cRGD/*Bmi-1RNAi*-PTX with 48h. i qRT-PCR results have showed that the cells have significantly reduced *CD133* gene expression ratio, we use one-way ANOVA exposure, Free PTX vs. other two groups, f(2)=1220.03, *p* < 0.001.

Flow cytometry showed that only trace amounts of CD133 were expressed in Hep-2 cells, with a surface membrane antigen expression of approximately 2.797 ± 1.017% (Figure 3a). As saw under an inverted microscope, CD133^+^ and CD133^-^ cells were successfully sorted and cultured at 2 × 10^3^ cells each well in a serum-free and low-adhesion 6-well cell culture plates. The CD133^+^ Hep-2 and CD133^-^ Hep-2 cells were all single, spherical and growing in suspension on the first day after sorting. We found that CD133^+^ Hep-2 cells were more easily enriched into spheres, was increasing in number and became larger with increasing days, while CD133^-^ Hep-2 cells were all dead on the fourth day (Figure 3b). The diameter of all cell spheres was measured from a single cell diameter of 24.283 ± 2.428 um on day 1 to a cell sphere diameter of 389.523 ± 26.667um on day 9 (Figure 3c). The sorted laryngeal carcinoma CD133^+^ Hep-2 cell population had strong clone formation and self-renewal ability, with the characteristics of CSCs.

C-myc, Nanog, Oct-4 and Sox-2 are all tumor-associated genes, and the core transcriptome of Nanog, Oct-4 and Sox-2 is involved in the self-renewal of CSCs. Hep-2 cells were incubated with Free PTX, mPEG-CS-cRGD/PTX and mPEG-CS-cRGD/*Bmi-1RNAi*-PTX for 48 h, the effects of mPEG-CS-cRGD/*Bmi-1RNAi*-PTX on the protein expression levels of C-myc, Nanog, Oct-4 and Sox-2 in Hep-2 cells were examined in Hep-2 cells. The results showed that the expression level of C-myc, Nanog, Oct-4 and Sox-2 proteins in Free PTX group, mPEG-CS-cRGD/PTX group and mPEG-CS-cRGD/*Bmi-1RNAi*-PTX group were gradually decreased, with corresponding C-myc, Nanog, Oct-4 and Sox-2 to 0.405 ± 0.027 (*p* < 0.001), 0.413 ± 0.020 (*p* < 0.001), 0.402 ± 0.020 (*p* < 0.001) and 0.408 ± 0.024 (*p* < 0.001). Followed by mPEG-CS-cRG/PTX group, that respectively to C-myc, Nanog, Oct-4 and Sox-2 decreased to 0.564 ± 0.032 (*p* < 0.001), 0.767 ± 0.033 (*p* < 0.001), 0.479 ± 0.038 (*p* < 0.001) and 0.513 ± 0.031 (*p* < 0.001) (Figure 3d). qRT-PCR results showed a consistent decrease in the western blot. Comparing to the Control group, mPEG-CS-cRGD/*Bmi-1RNAi*-PTX mRNA expression corresponding to C-myc was reduced to 0.405 ± 0.011 (*p* < 0.01), Nanog to 0.428 ± 0.010 (*p* < 0.001), Oct-4 to 0.396 ± 0.011 (*p* < 0.001), and Sox-2 to 0.393 ± 0.012 (*p* < 0.001) (Figure 3e).

In this study, we used the chemotherapeutic drug PTX to enrich laryngeal CSCs, and Western blotting was utilized to detect the expression levels of CSCs markers in Hep-2 cells after control and chemotherapy enrichment. The highest protein expression was 0.896 ± 0.013, while the PTX-resistant cell population showed increased protein expression of C-myc, Nanog, Oct-4, Sox-2 and CD133, 1.043 ± 0.047 (*p* < 0.001), 1.020 ± 0.020 (*p* < 0.001), and 1.024 ± 0.026 (*p* < 0.001), 1.004 ± 0.021 (*p* < 0.01) and 1.022 ± 0.057 (*p* < 0.001) respectively (Figure 3f). qRT-PCR results showed that gene expression was respectively consistent with protein expression, and compared to pre-enrichment, post-enrichment expression of C-myc, Nanog, Oct-4, Sox-2 and CD133 were 2.076 ± 0.019 (*p* < 0.001), 1.274 ± 0.082 (ns), 1.772 ± 0.155 (*p < 0*.05), 1.499 ± 0.167 (*p < 0*.05) and 1.621 ± 0.016 (*p* < 0.001) after enrichment compared to before (Figure 3g).

CD133 is part of the markers of laryngeal CSCs. To confirm that mPEG-CS-cRGD/*Bmi-1RNAi*-PTX can target laryngeal CSCs and decrease the expression of markers related to laryngeal CSCs, Hep-2 cells enriched with PTX chemotherapy were treated with mPEG-CS-cRGD/PTX and mPEG-CS-cRGD/*Bmi-1RNAi*-PTX for 48h. After treatment with mPEG-CS-cRGD/PTX, the expression levels of CD133 protein levels were observed, and it was seen that mPEG-CS-cRGD/*Bmi-1RNAi*-PTX reduced the expression of CD133 protein in Hep-2 cells. CD133 expression of mPEG-CS-cRGD/PTX group was 0.728 ± 0.005 (*p* < 0.001), while mPEG-CS-cRGD/*Bmi-1RNAi*-PTX was only 0.566 ± 0.040 (*p* < 0.001) (Figure 3h). Meanwhile qRT-PCR results showed that the Hep-2 cells treated with mPEG-CS-cRGD/PTX, CD133 gene expression rate was 0.605 ± 0.007 (*p* < 0.001), while mPEG-CS-cRGD/*Bmi-1RNAi*-PTX was significantly reduced, corresponding to an expression rate of 0.338 ± 0.017 (*p* < 0.001) (Figure 3i).

### Effect of mPEG-CS-cRGD/Bmi-1RNAi-PTX on the migration ability of Hep-2 cells

**Figure 4.**
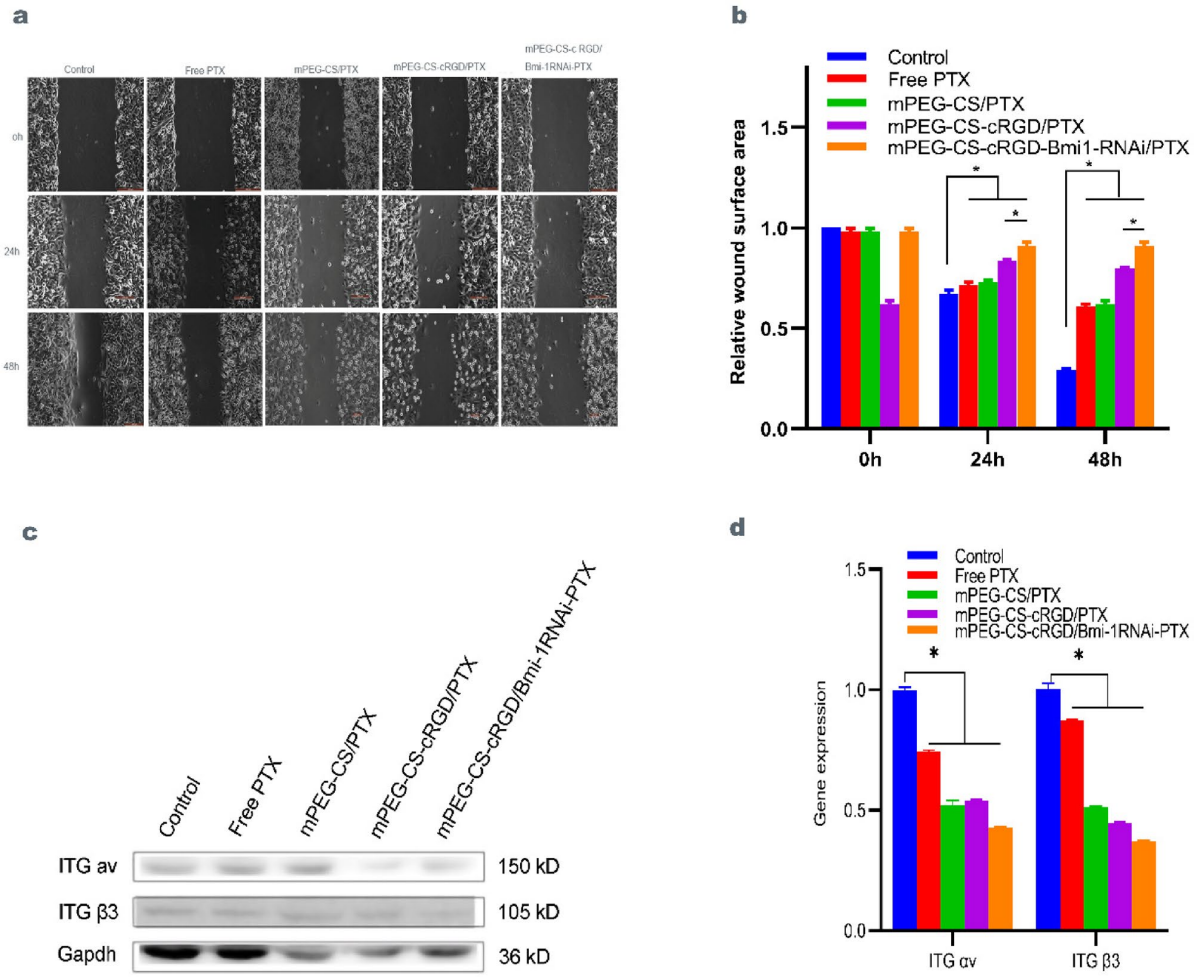
a mPEG-CS-cRGD/*Bmi-1RNAi*-PTX had the slowest wound healing space and cell growth rate. b The wound healing area at 48h had the smallest change compared with 0h and 24h, we use one-way ANOVA exposure, control vs. other four groups, 24h: f(4)=136.53, *p* < 0.01. 48h: f(4)=1468.83, *p* < 0.001. c mPEG-CS-cRGD/*Bmi-1RNAi*-PTX reduced the expression of ITG αv and ITG β3. d The qRT-PCR results were consistent with western blotting we use one-way ANOVA exposure, control vs. other four groups, ITG αv: f(4)=1024.49, *p* < 0.001. ITG β3: f(4)=2301.82, *p* < 0.001.

The scratch healing assay examined the effect of mPEG-CS-cRGD/*Bmi-1RNAi*-PTX on the migration of Hep-2 cells. The results showed that the healing distance became smaller with time migration, and the mPEG-CS-cRGD/*Bmi-1RNAi*-PTX group had the largest scratch healing distance opposed with the other four groups, which effectively inhibited the migration of Hep-2 cells. The strong effects of the four treatment groups on the migration ability of Hep-2 cells were: Free PTX > mPEG-CS/PTX > mPEG-CS-cRGD/PTX > mPEG-CS-cRGD/*Bmi-1RNAi*-PTX (Figure 4a ∼ b). Being compared to the Control group, the other four groups after treatment were statistically different at 24 h and 48 h (*p* < 0.001). We use western blot test to check whether mPEG-CS-cRGD/*Bmi-1RNAi*-PTX reduced the expression of ITG αvβ3 protein. The result showed that, comparing to the Control group, the Free PTX group and mPEG-CS/PTX groups had no difference in ITG αv and ITG β 3 in the Free PTX and mPEG-CS/PTX groups. The protein expression of ITG αv and ITG β3 in the Free PTX group was respectively 1.017 ± 0.020 (ns) and 0.879 ± 0.029 (*p* < 0.001), while in the mPEG-CS/PTX group it was 1.002 ± 0.020 (ns) and 0.905 ± 0.030 (*p < 0*.01), and the Hep-2 cells treated with mPEG-CS-cRGD/PTX started to show a significant decrease in protein expression of ITGαv at 0.544 ± 0.007 (*p* < 0.001) and mPEG-CS-cRGD/Bmi-1RNAi-PTX group was 0.560 ± 0.007 (*p <* 0.001). ITG β3 also showed the above pattern of alteration, with ITG β3 protein expression in the mPEG-CS-cRGD/PTX and mPEG-CS-cRGD/Bmi-1RNAi-PTX groups being 0.718 ± 0.015 (*p* < 0.001) and 0.519 ± 0.015 respectively (*p* < 0.001), which showed a smaller change compared to ITG αv (Figure 4c). qRT-PCR and western blotting results were consistent. Comparing to the Control group, the mRNA expression of ITG αv in Free PTX, mPEG-CS/PTX, mPEG-CS-cRGD/PTX and mPEG-CS-cRGD/*Bmi-1RNAi*-PTX was respectively with 0.742 ± 0.005 (*p <* 0.001), 0.521 ± 0.016 (*p* < 0.001), 0.540 ± 0.003 (*p* < 0.001) and 0.428 ± 0.002 (*p* < 0.001). And ITG β3 were respectively with 0.871 ± 0.005 (*p* < 0.001), 0.512 ± 0.004 (*p* < 0.001), 0.448 ± 0.003 (*p* < 0.001) and 0.372 ± 0.002 (*p* < 0.001). Thus ITG αv and ITG β3 were both statistically different (*p <* 0.001) (Figure 4d). It demonstrated that mPEG-CS-cRGD/*Bmi-1RNAi*-PTX not only affected the growth of Hep-2 cells, but also decreased the ITG αvβ3 expression, thereby inhibiting the migratory ability of Hep-2 cells.

### Effect of mPEG-CS-cRGD/Bmi-1RNAi-PTX on apoptosis of Hep-2 cells

**Figure 5.**
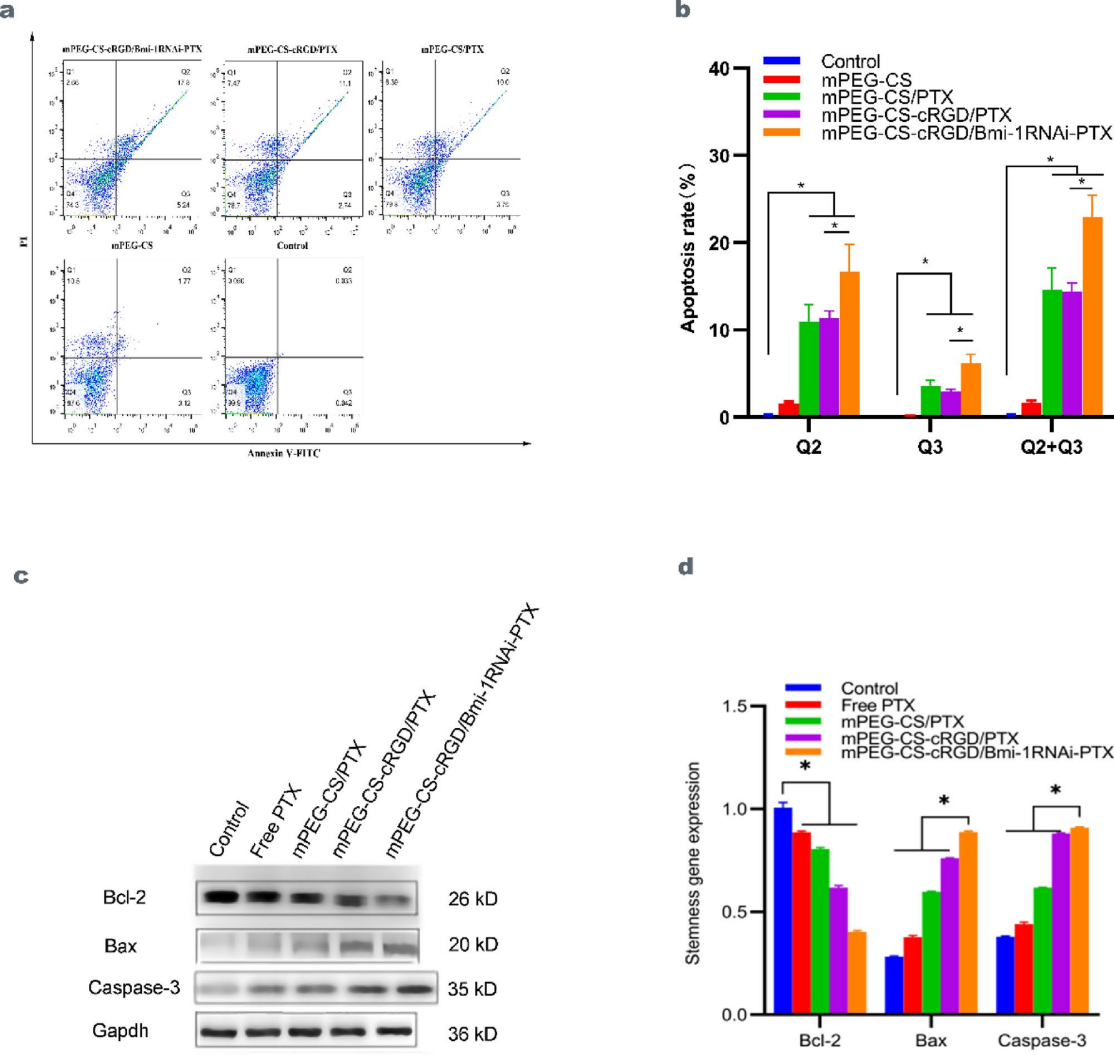
a Compared with the control group, Free PTX, mPEG-CS/PTX, mPEG-CS-cRGD/PTX and mPE-CS-cRGD/*Bmi-1RNAi*-PTX group had good apoptosis-inducing effect on cells. b We use one-way ANOVA exposure, control vs. other three groups, Q2: f(4)=53.68, *p* < 0.001. Q3: f(4)=72.54, *p* < 0.001. Q2 + Q3: f(4)=106.76, *p* < 0.001. c Western blot results showed that the protein expression levels of Bcl-2, Bax and Caspase-3 were significantly increased in Hep-2 cells treated with mPEG-CS-cRGD/*Bmi-1RNAi*-PTX. d The results were consistent with qRT-PCR, we use one-way ANOVA exposure, control vs. other four groups, Bcl-2: f(4)=1003.76, *p* < 0.001. Bax: f(4)=9697.58, *p* < 0.001. Caspase-3: f(4)=6499.82, *p* < 0.001.

The effect of mPEG-CS-cRGD/*Bmi-1RNAi*-PTX on Hep-2 cells was examined by Annexin V-FITC flow-through apoptosis kit. Q2 indicates early apoptosis, Q3 indicates late apoptosis and necrosis, and Q2 + Q3 indicates the proportion of apoptosis cells. Chromatin condensation in the nuclei of apoptosis cells produced bright apoptosis vesicles. The mPEG-CS/PTX, mPEG-CS-cRGD/PTX and mPE-CS-cRGD/*Bmi-1RNAi*-PTX groups all had a strong pro-apoptotic effect on Hep-2 cells relative to the Control and mPEG-CS group. Among them, mPEG-CS-cRGD/*Bmi-1RNAi*-PTX had the largest effect, and there was a statistical difference (*p* < 0.001) in Q2, Q3 and Q2 + Q3 relative to the Control and mPEG-CS group (Figure 5a ∼ b). From the experimental data, we can know that, compared to the Control and mPEG-CS group, mPEG-CS-cRGD/*Bmi-1RNAi*-PTX acting on Hep-2 cells, the protein and mRNA expression of anti-apoptosis-related genes Bcl-2 were substantially reduced, with 0.546 ± 0.002 (*p* < 0.001) and 0.403 ± 0.005 (*p* < 0.001). While the Bax and Caspase-3 were substantially increased, with respectively protein and mRNA expression of Bax at 1.003 ± 0.02 (*p* < 0.001) and 1.014 ± 0.015 (*p* < 0.001). And Caspase-3 at 1.008 ± 0.025 (*p* < 0.001) and 1.012 ± 0.016 (*p* < 0.001). What is more, mRNA expression of both Bax (*p* < 0.001) and Caspase-3 (*p* < 0.001) was statistically different reported to the Control and mPEG-CS group (Figure 5c ∼ d).

### Suppressive effect of mPEG-CS-cRGD/Bmi-1RNAi-PTX on mouse laryngeal carcinoma transplantation tumors

**Figure 6.**
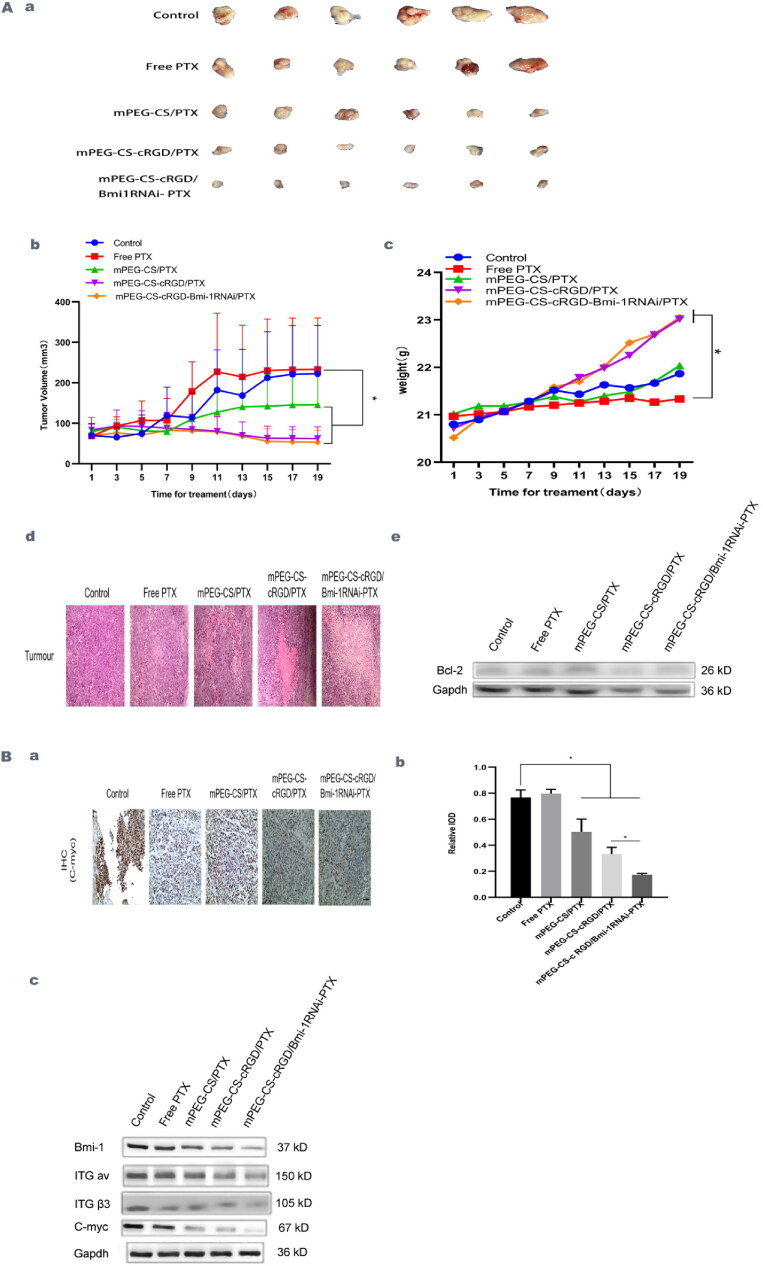
**A.** Suppressive effect of mPEG-CS-cRGD/*Bmi-1RNAi*-PTX on mouse laryngeal carcinoma transplantation tumors. **a** Tumor volume changes in mice. b We use one-way ANOVA exposure, control vs. other three groups, f(4)=19.12, *p* < 0.05. c Changes in body weight of mice, we use one-way ANOVA exposure, control vs. two groups, f(4)=6.377, *p* < 0.01. d H&E staining of mice tumor(200x). e The protein level of Bcl-2 by western blot. B mPEG-CS-cRGD/*Bmi-1RNAi*-PTX effectively inhibited Bmi-1 expression in vivo and reduced the protein expression levels of ITG αv, ITGβ3, C-myc, Nanog, Oct-4 and Sox-2. **a** Immunohistochemical analysis of the mice tumor tissue of C-myc. b We use one-way ANOVA exposure, control vs. other three groups, f(4)=64.38, *p* < 0.001(200x). c The protein level of Bmi-1, ITG αv, ITG β3 and C-myc by western blot.

BALB/c-nude mice were used as a laryngeal cancer animal model to observe the inhibition of laryngeal cancer cells by nanomedicine particles in vivo. When the subcutaneous tumor volume of the mice reached approximately 300 mm^3^, mPEG-CS/PTX, mPEG-CS-cRGD/PTX and mPEG-CS-cRGD/*Bmi-1RNAi*-PTX were administered intravenously with Free PTX, mPEG-CS-cRGD/PTX and mPEG-CS-cRGD/*Bmi-1RNAi*-PTX tails respectively. After 19 days of intravenous treatment administration, the tumor volume of the Control group, the Free PTX group and the mPEG-CS/PTX group were increased slowly, while the tumor volume of the mPEG-CS-cRGD/PTX group and the mPEG-CS-cRGD/*Bmi-1RNAi*-PTX group were declined. There was a significant difference compared with the Control group, while the mice injected with mPEG-CS-cRGD/*Bmi-1RNAi*-PTX had the most inhibited tumor tissues and no significant regeneration (*p* < 0.001) (Figure 6Aa ∼ b). In addition, the weight changes of nude mice during 19 days of treatment were observed. The further statistical analysis showed that compared with the Control group, the weight of nude mice in the mPEG-CS-cRGD/PTX group and the mPEG-CS-cRGD/*Bmi-1RNAi*-PTX group were significantly increased (*P* < 0.01), the body weight were 23.017 ± 0.349 mg and 23.050 ± 0.476 mg, indicating that within a certain concentration range of nano-complex (PTX:5 mg/kg, *Bmi-1RNAi* 10 ug of each mice), there was no obvious toxic and side effect on nude mice (Figure 6Ac). The systemic toxic effects of nano-drug particles on mice were further assessed by analyzing organ tissue sections for each group of administered mice (Supplementary Figure 2). Tumor tissues from the 5 groups were excised, H&E stained to observe the cell morphology of the Control group, the tumor tissues of the Control group showed a uniform distribution of cancer cells with obvious heterogeneity, and most of them showed punctate necrosis, while the tumor tissues of mPEG-CS/PTX, mPEG-CS-cRGD/PTX and mPEG-CC-cRGD/*Bmi-1RNAi*-PTX showed an increasing degree of necrosis compared with the Free PTX group. Among them, the mPEG-CC-cRGD/*Bmi-1RNAi*-PTX group showed obvious large patchy necrosis, and even a part of them showed map-like changes (Figure 6Ad). Compared with the Control group, the anti-apoptotic protein Bcl-2 was found in Free PTX, mPEG-CS/PTX, mPEG-CS-cRGD/PTX and mPEG-CS-cRGD/*Bmi-1RNAi*-PTX showed a respectively progressive decrease in protein expression of 1.014 ± 0.015 (ns), 1.012 ± 0.015 (ns), 0.602 ± 0.013 (*p* < 0.001) and 0.445 ± 0.022 (*p* < 0.001) (Figure 6Ae), which was consistent with the results of in vitro experiments. Further confirmed that mPEG-CC-cRGD/*Bmi-1RNAi*-PTX could be targeted and deposited in tumor tissues in mice, markedly inhibiting the growth of transplanted tumor cells and prompting their apoptosis.

### mPEG-CS-cRGD/Bmi-1RNAi-PTX effectively inhibited Bmi-1 expression in vivo and reduced the protein expression levels of ITG αv, ITGβ3, C-myc, Nanog, Oct-4 and Sox-2

To further verify that mPEG-CS-cRGD/*Bmi-1RNAi*-PTX can inhibit the growth of laryngeal cancer tissues and reduce the invasive metastasis of laryngeal cancer in vivo by reducing the expression of genes related to laryngeal CSCs, immunohistochemical analysis of laryngeal cancer xenograft tumors was performed with C-myc antibody, and the results showed that consistent with the in vitro results, compared with the Control group, C-myc expression of Free PTX group showed a small increase, while mPEG-CS/PTX, mPEG-CS-cRGD/PTX and mPEG-CS-cRGD/*Bmi-1RNAi*-PTX showed a stepwise decrease, and the data statistics showed a statistical difference (*p* < 0.001) (Figure 6Ba ∼ b). In vivo treated with Free PTX, mPEG-CS/PTX, mPEG-CS-cRGD/PTX and mPEG-CS-cRGD/*Bmi-1RNAi*-PTX, protein of the mouse tumor tissues expression levels of *Bmi-1* and C-myc were gradually reduced compared to the Control group, and the protein expression of *Bmi-1 in the* four groups were 0.899 ± 0.0169 (*p* < 0.001), 0.827 ± 0.020 (*p* < 0.001), 0.645 ± 0.016 (*p* < 0.001) and 0.406 ± 0.010 (*p* < 0.001) for *Bmi-1*. Then the C-myc in the four groups were respectively with 0.913 ± 0.018 (*p* < 0.001), 0.648 ± 0.013 (*p* < 0.001), 0.447 ± 0.009 (*p* < 0.001) and 0.358 ± 0.007 (*p* < 0.001), which were consistent with the in vitro results, demonstrating that mPEG-CS-cRGD/*Bmi-1RNAi*-PTX was also effective in knocking down *the Bmi-1 gene* in vivo, reducing the laryngeal expression of the stemness marker of C-myc in CSCs and inhibited the growth of transplanted tumors. The expression levels of ITG αv and ITG β3 were also gradually reduced in the Free PTX group, mPEG-CS/PTX group, mPEG-CS-cRGD/PTX group and mPEG-CS-cRGD/*Bmi-1RNAi-PTX* group, where mPEG-CS-cRGD/*Bmi-1RNAi*-PTX showed the greatest decrease in protein expression, with the protein expression of ITG αv and ITG β3 in mPEG-CS-cRGD/*Bmi-1RNAi*-PTX compared to the Control group at 0.423 ± 0.008 (*p* < 0.001) and 0.411 ± 0.004 (*p* < 0.001) (Figure 6Bc). It was suggested that mPEG-CS-cRGD/*Bmi-1RNAi*-PTX significantly inhibited the growth of mouse laryngeal carcinoma transplantation tumors, effectively suppressed the expression of ITGαv and ITGβ3 in vivo and reducing the local invasion of tumors. mPEG-CC-cRGD/*Bmi-1RNAi*-PTX targeted tumor tissues in vivo in mice, down-regulated the expression of *Bmi-1* and made tumor cells to be more sensitive to PTX. At the same time, directing PTX targeting to the target tumor cells, overcoming the shortcomings of PTX resistance, not easy to lyse and easy to damage normal tissue cells, to achieve synergistic targeting of laryngeal cancer to treat CSCs.

## Discussion

For patients with advanced laryngeal cancer, the current treatment is always based on a combination of surgical treatment and radiotherapy as an adjunct (Zhu et al., 2020). However, the recurrence and metastasis of laryngeal cancer after conventional treatment and resistance to radiotherapy are still major problems that are addressed urgently, and new therapeutic approaches are urgently needed. In this study, a nanocomplex particle, mPEG-CS-cRGD/*Bmi-1RNAi*-PTX, which can multi-target laryngeal CSCs, was synthesized. It is one of the ideal target drugs for laryngeal cancer.

Polymeric nanoparticles are nanotechnology applied to nanomedicine (Wathoni et al., 2019). mPEG-CS NPs as one of the well-established nanocarriers, CS can be regenerated and recycled, as they are not easily soluble and are readily removed by in vivo biological clearance barriers, resulting in CS not being applied singularly in vivo. Experiments have shown that mPEG-CS nanocarriers formed after mPEG modification was more soluble in body fluids and have prolonged blood circulation time (Helmi et al., 2021). The nanopores or nanochannels are stimulated by external PH factors, leading to changes in ion transport properties, i.e., PH responsiveness, the mPEG-CS NPs are PH-responsive NPs, which to a certain extent achieve a controlled release of the substances they carry (Ait Bachir et al., 2018). The EPR effect can be used to passively target the target tissues, which can cross the capillaries, tissue gaps and endothelial cells to release the drug at the cellular or subcellular level, whereas the micro vascular endothelial cell gaps in normal tissues are less than 10 nm, which cannot be crossed by NPs, which also provides the possibility for NPs to transport anti-cancer drugs into tumor tissues, and is the basis for their tumor targeting properties (Liu et al., 2016). The difference between the vasculature of normal tissues and the neovascularization of tumor tissues lies in the leakiness of the latter, with the majority of tumor vessels having "small pores"(3 8 0 ∼ 780 nm) (Xin et al., 2016). In this study, the size of our synthesized mPEG-CS-cRGD/*Bmi-1RNAi*-PTX was approximately 182 ± 30.6 nm (Table 1) (Figure 1Ba). Therefore, mPEG-CS can carry genes, PTX and cRGD peptides in order to easily cross the cell membrane and get into the cell.

CD133 cells have stronger proliferation, invasion, metastasis and self-renewal capabilities, and have CSCs biological properties, which are key factors leading to recurrence, metastasis, and resistance to radiotherapy and chemotherapy in laryngeal cancer (Silva Galbiatti-Dias et al., 2018) (Figure 3a ∼ c). C-myc is located on chromosome 8q24, as one of the Myc family, is one of the genetic markers of the CSCs cluster and one of the proto-oncogenes that can be induced to acquire CSCs by inducing epigenetic coding (Yoshida, 2018). It is also engaged in tumor cell proliferation cycle changes, chemoresistance, apoptosis and other processes (Caforio et al., 2018). Oct-4 has been linked to laryngeal cancer staging and lymphatic metastasis (El Deeb & Abdelzaher, 2014). In CSCs, overexpression of the transcription factor Nanog, in coordination with Oct-4, Sox-2, Bmi-1 and other complexes, enhanced the characteristic expression of CSCs and activated their self-renewal, metastasis, invasion, angiogenesis and drug resistance, thereby reducing tumor cell apoptosis (Najafzadeh et al., 2021). Previous reports have shown that C-myc (Tian et al., 2018), Oct-4 (El Deeb & Abdelzaher, 2014), Nanog and Sox-2, as biomarkers of CSCs, have not been reported to be associated with laryngeal cancer. The results of this experiment showed that mPEG-CS-cRGD/*Bmi-1RNAi*-PTX co-cultured with laryngeal Hep-2 cells effectively killed laryngeal CSCs, while the markers C-myc, Nanog, Oct-4, Sox-2 and the proven marker CD133 of laryngeal CSCs were reduced together, and it could be predicted that Nanog and Sox-2 may also be candidate markers for laryngeal CSCs in vitro (Figure 3d ∼ e). In vivo, C-myc expression was similarly reduced in protein and gene expression (Figure 6B). Compared with tumor sphere enrichment and CSCs marker sorting, chemotherapy drug enrichment is simple and inexpensive, and prevents the need for a single marker to affect the purity of the cells after sorting, as well are closer to the real CSCS (Weiss et al., 2021). In vitro, Cells enriched with PTX of C-myc, Nanog, Oct-4 and Sox-2 in laryngeal Hep-2 cells, all showed a decreasing trend (Figure 3f ∼ g). According to the theory of CSCs, after several mitotic divisions following the action of chemotherapeutic drugs, most tumor cells undergo apoptosis, while CSCs are mostly in quiescent phase, thus escaping the killing effect of chemotherapeutic drugs and experiencing recurrence and metastasis at the end of chemotherapy (Walcher et al., 2020). We observed that mPEG-CS-cRGD/*Bmi-1RNAi*-PTX significantly reduced the mRNA transcript and protein expression levels of CD133 in laryngeal cancer Hep-2 cells compared with the Free PTX group (*p* < 0.001) in vitro.(Figure 3h ∼ i). Therefore, mPEG-CS-cRGD/*Bmi-1RNAi*-PTX can target and suppressed laryngeal cancer by targeting laryngeal CSCs and reduce the generation of chemotherapy resistance to PTX.

The passive targeting of mPEG-CS nanocarriers combined with the active targeting of cRGD peptide and *Bmi-1RNAi* exerts a synergistic effect to enhance the chemosensitivity and reduce the drug resistance of PTX. mPEG-CS-cRGD/Bmi-1RNAi-PTX killed almost all Hep-2 cells and mice laryngeal carcinoma transplant as confirmed by cck-8 proliferation-toxicity assay (Figure 2b), colony formation assay (Figure 2c ∼ d) and H&E staining (Fig. 6Aa ∼ b). Bcl-2 is a family of apoptosis proteins, to be the main regulator of apoptosis, and Bax is an effector of Bcl-2 apoptosis, which has an anti-apoptosis effect and a pro-apoptosis effect during tumorigenesis. Increasing Bcl-2/Bax ratio can promote the caspase cascade and programmed cell death (García-Aranda et al., 2018). The mPEG-CS-cRGD/*Bmi-1RNAi*-PTX could activate the internal apoptosis pathway (Caspase-3) in Hep-2 cells, altering mitochondrial permeability and further aggravating tissue damage (Figure 5c ∼ d, 6Ae). The above results showed that, especially in vivo, mPEG-CS-cRGD/*Bmi-1RNAi*-PTX had a stronger killing effect on Hep-2 cells and the mice laryngeal cancer tissues than Free PTX, which could effectively reduce the proliferation and increase the apoptosis of Hep-2 cells and mouse laryngeal cancer tissues (Figures 2–6), which was related to the active targeting and passive targeting effects of NPs. In vivo experiments, 19 days after drug intervention, we can see that the final synthesized nanocomplex has basically the same effect as the complex without Bmi-1 gene knockdown.It may be due to the small volume of tumor formation, and the two groups of drugs can effectively inhibit the growth of tumor in nude mice, leading to the insufficient effectiveness gap in the course of short-term action (6 times). We can see that after 19 days of drug intervention, the tumor tissue only becomes smaller, but does not disappear. Based on the 19-day post-action inhibition results, we concluded that, if the experimental conditions are increased, the tumor tissue be completely eliminated.

In this experiment, NPs were injected into the tail vein of tumor-bearing mice to observe the therapeutic effect of nanomedicine as an example on laryngeal cancer. It was demonstrated that the mPEG-CS-cRGD/*Bmi-1RNAi*-PTX nanocomplex prepared in this study had good effects in inhibiting the growth of Hep-2 cells in vitro and inhibiting the growth of mouse laryngeal cancer transplantation tumors in vivo, but the mPEG-CS-cRGD/Bmi-1RNAi-PTX nanocomplexes in vivo, we need to further investigate how they are distributed and metabolism in vivo.

## Conclusion

In this study, mPEG-CS-cRGD/*Bmi-1RNAi*-PTX NPs were successfully prepared with a spherical shape, a particle size of 182 ± 30.6 nm, a zeta potential of 10.14 ± 0.52 MV, an encapsulation rate of 84.84% and a drug loading capacity of 4.24%, which have good protection for the loaded genes, long circulation capacity and high drug loading capacity. It is an ideal particle for gene therapy because of its extensive circulation capacity and high loading capacity and encapsulation rate. The nanocomplexes are effective in transfection and are ideal for knocking out the *Bmi-1* gene. The nanocomplex can target and transfect laryngeal CSCs in vivo and in vitro, silencing the *Bmi-1* gene and achieving precise chemotherapy with PTX. The proliferation and differentiation of laryngeal CSCs after transfection with the nanocomplex decreased in vitro and apoptosis increased. In vivo tumorigenicity was significantly reduced and tumor suppression was remarkable. The combination of knockdown of *Bmi-1* gene, precise chemotherapy with PTX and cRGD-targeted peptide could have a significant impact on the overlapping gene therapy of laryngeal CSCs. The combination of *Bmi-1* knockdown, PTX precision chemotherapy and cRGD targeting peptide had overlapping and potent killing effects on laryngeal CSCs. This nano-complex is expected to be a novel, safe and effective nano-drug for multi-targeting laryngeal CSCs.

## Supplementary Material

Supplemental Material
