# Supplementary figures and images for: Application of mPEG-CS-cRGD/Bmi-1RNAi-PTX nanoparticles in suppression of laryngeal cancer by targeting cancer stem cells
Source: Drug Deliv. 2023 Feb 21;30(1):2180112. doi: 10.1080/10717544.2023.2180112 (PMC9946312; doi:10.1080/10717544.2023.2180112)

**Supplementary Fig 1**


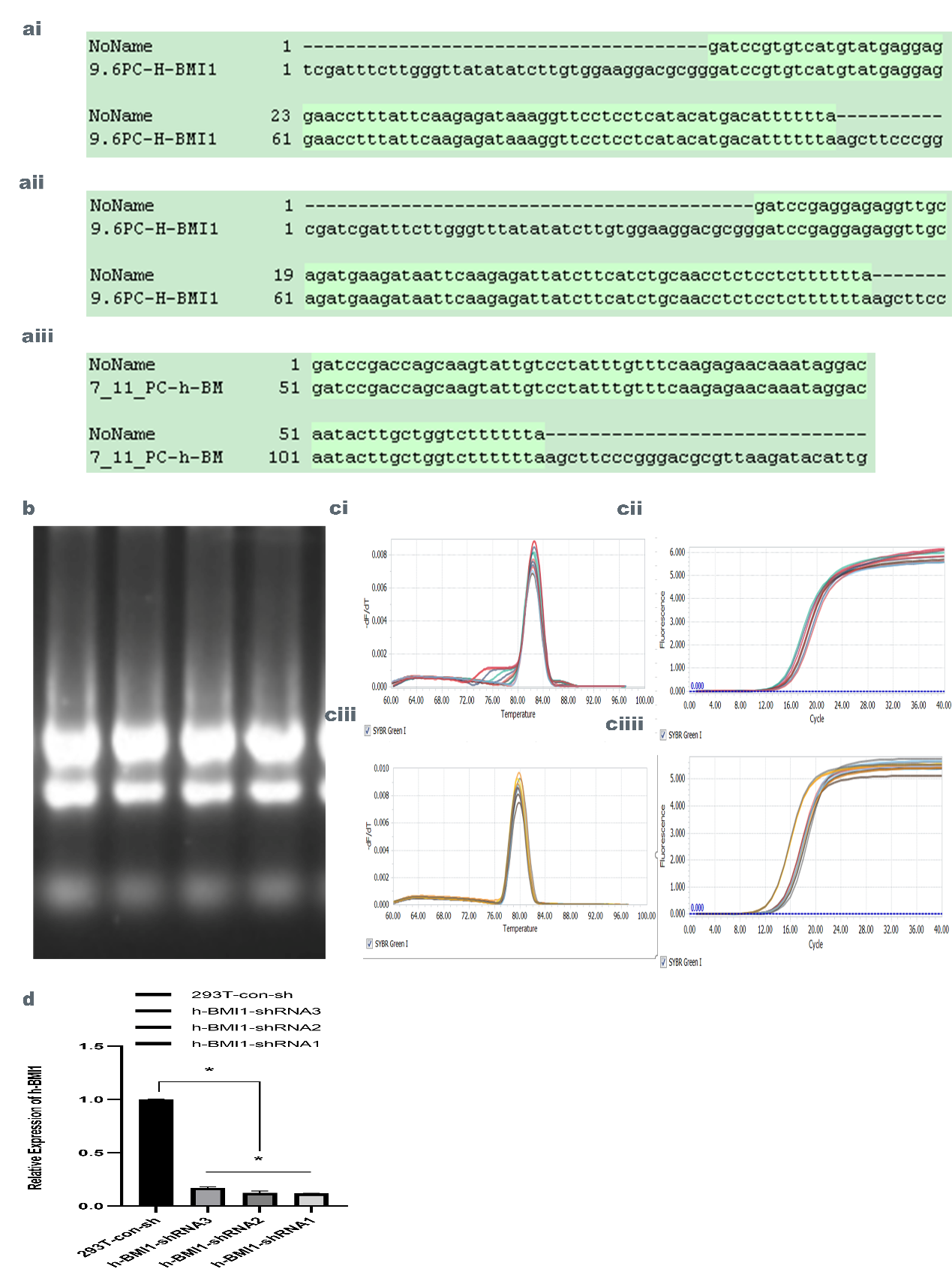


**Supplementary Fig. 2**


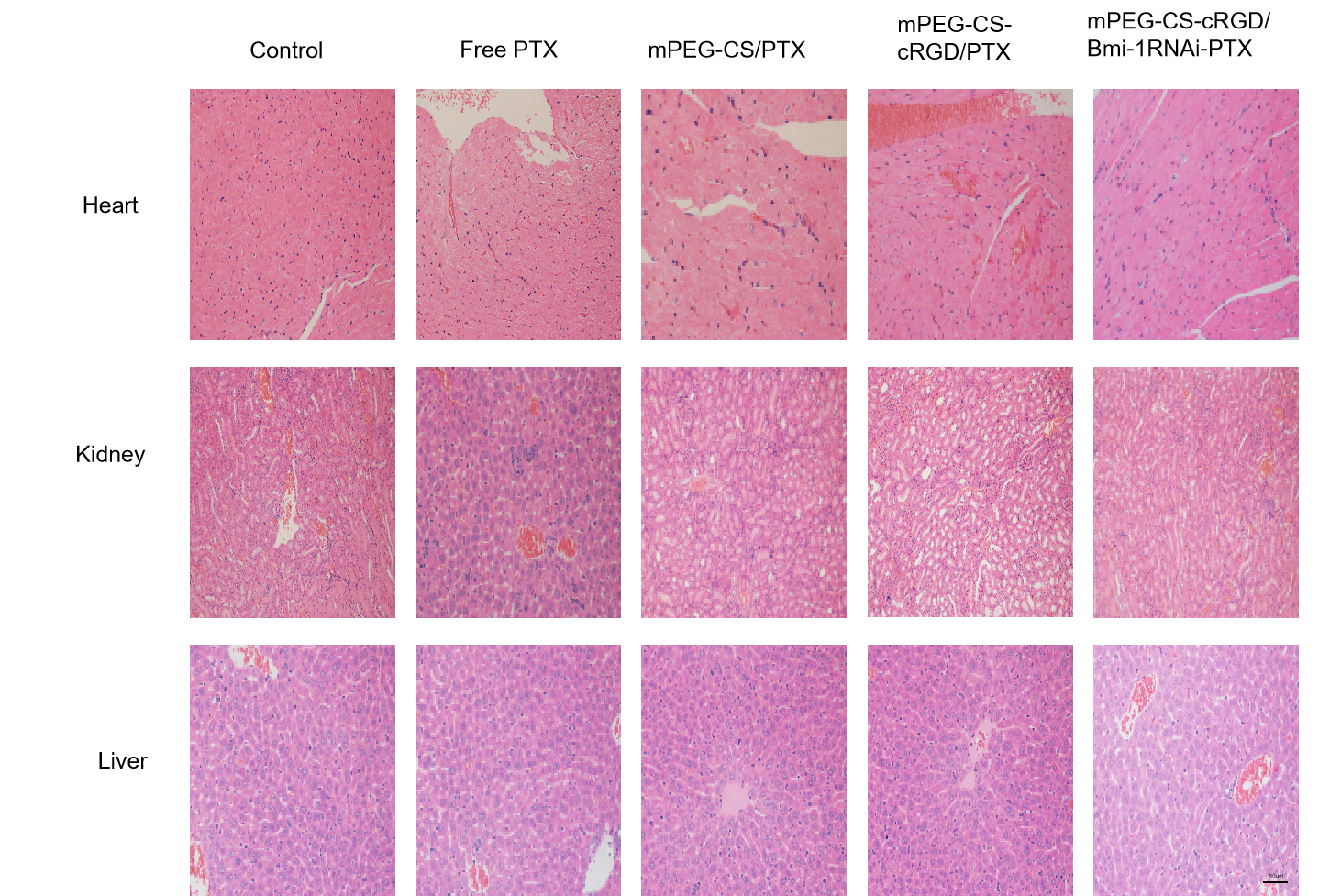

Supplement: Supplemental Material [file IDRD_A_2180112_SM6224.docx]
